# Immunofluorescence studies to dissect the impact of Cockayne syndrome A alterations on the protein interaction and cellular localization

**DOI:** 10.1186/s43141-021-00190-7

**Published:** 2021-06-16

**Authors:** Amr Ghit

**Affiliations:** 1 Department of Biology and Biotechnology, University of Pavia, Pavia, Italy; 2 Department of Biotechnology, Institute of Graduate Studies and Research (IGSR), Alexandria University, Alexandria, Egypt

**Keywords:** Cockayne syndrome, TRiC/CCT complex, Nucleotide excision repair, CSA, CSB, Immunofluorescence

## Abstract

**Background:**

Cockayne syndrome (CS), which was discovered by Alfred Cockayne nearly 75 years ago, is a rare autosomal recessive disorder characterized by growth failure, neurological dysfunction, premature aging, and other clinical features including microcephaly, ophthalmologic abnormalities, dental caries, and cutaneous photosensitivity. These alterations are caused by mutations in the CSA or CSB genes, both of which are involved in transcription-coupled nucleotide excision repair (TC-NER), the sub-pathway of NER that rapidly removes UV-induced DNA lesions which block the progression of the transcription machinery in the transcribed strand of active genes. Several studies assumed that CSA and CSB genes can play additional roles outside TC-NER, due to the wide variations in type and severity of the CS phenotype and the lack of a clear relationship between genotype and phenotype. To address this issue, our lab generated isogenic cell lines expressing wild type as well as different versions of mutated CSA proteins, fused at the C-terminus with the Flag and HA epitope tags (CSA^Flag-HA^). In unpublished data, the identity of the CSA-interacting proteins was determined by mass spectrometry. Among which three subunits (namely, CCT3, CCT8, and TCP1) of the TRiC/CCT complex appeared as novel interactors. TRiC is a chaperonin involved in the folding of newly synthesized or unfolded proteins. The aim of this study is directed to investigate by immunofluorescence analysis the impact of the selected CSA mutations on the subcellular localization of the CSA protein itself as well as on its novel interactors CCT3, CCT8, and TCP1.

**Results:**

We showed that specific CSA mutations impair the proper cellular localization of the protein, but have no impact on the cellular distribution of the TRiC subunits or CSA/TRiC co-localization.

**Conclusion:**

We suggested that the activity of the TRiC complex does not rely on the functionality of CSA.

**Supplementary Information:**

The online version contains supplementary material available at 10.1186/s43141-021-00190-7.

## Background

**Fig. 1:**
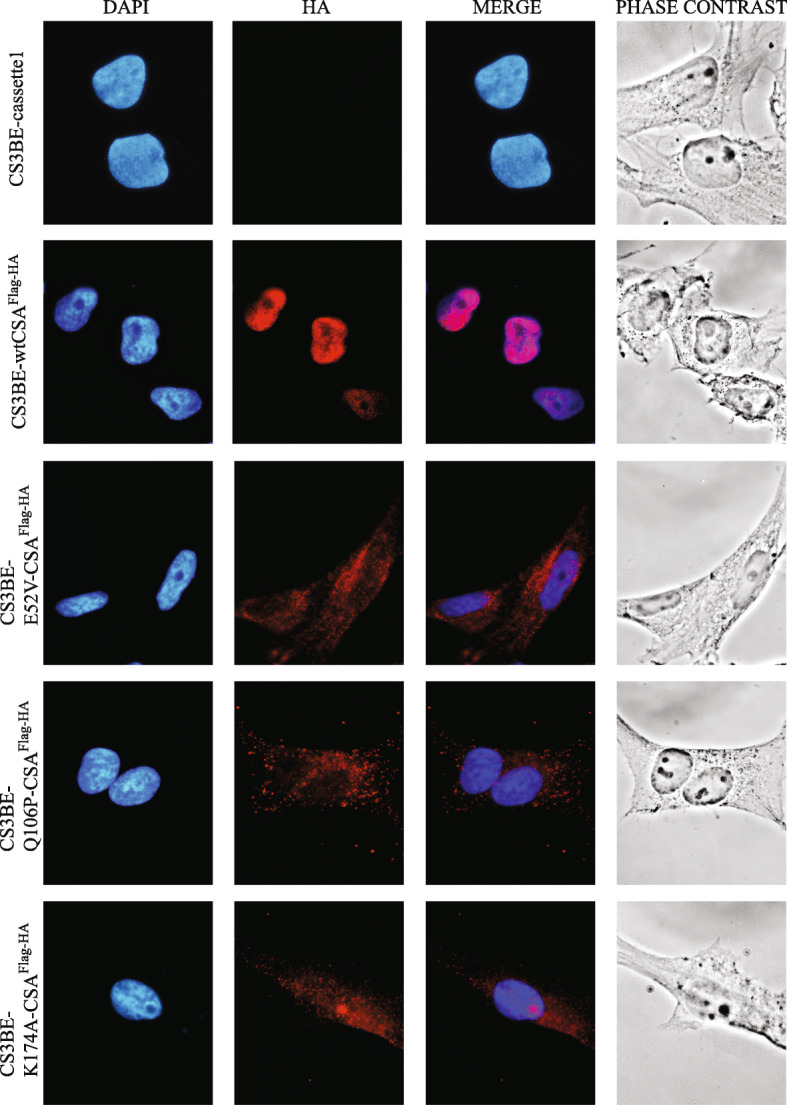
Subcellular localization of the recombinant CSA^Flag-HA^ proteins. Immunofluorescence staining with anti-HA antibodies (*red*) in CS3BE-cassette1, CS3BE-wtCSA^Flag-HA^, CS3BE-E52V-CSA^Flag-HA^, CS3BE-Q106P-CSA^Flag-HA^, and CS3BE-K174A-CSA^Flag-HA^. Nuclei were counterstained with DAPI (*blue*)

Cockayne syndrome (CS) is a rare autosomal recessive disorder characterized by growth retardation, neurodevelopmental abnormalities, premature aging, and cutaneous photosensitivity [1]. These alternations due to mutations in either the *CSB* or the *CSA* gene [2] which are key players in transcription-coupled nucleotide excision repair (TC-NER), the sub-pathway of NER that rapidly removes DNA helix-distorting lesions blocking the progression of the transcription machinery in the transcribed strand of active genes [3]. Differently, the NER sub-pathway called global genome repair (GGR) removes DNA damage from the silent regions of the genomes and is unaffected in CS [4]. The role of CSA and CSB proteins in TC-NER has been well characterized [5–8]. Nevertheless, the wide range of phenotypic manifestations as well as the lack of clear genotype-phenotype relationships emerged from mutational analysis in CS patients has further supported the notion that CS proteins might have additional functions outside TC-NER. In particular, the lack of skin cancer despite the persistency of DNA damage as well as the premature aging coupled with neurological deterioration are suggestive of transcriptional impairment and/or accumulation of oxidative damage [9]. In vivo and in vitro studies lead to demonstrate that CSA and CSB are not only involved in the repair of UV-induced DNA damage but also in the removal of oxidative DNA lesions [10]. Furthermore, CSA and CSB proteins appear involved in gene expression regulation and chromatin remodeling [11] as well as redox balance and cellular bioenergetics [12]. CSA protein, which belongs to the large WD repeat family, is consisting of 396 amino acids [13]. WD repeats are domains made up of about 40 amino acids that end with a dipeptide, tryptophan-aspartic acid (W-D), at the C-terminus [14, 15]. CSA contains seven WD repeats which create stable interactions with various partners [16]. Therefore, the identification of proteins and protein complexes interacting with CSA is a promising strategy to unravel the still unidentified functions of CSA, which might be relevant to define the genotype-phenotype relationship of CS patients. This study is a part of a broader project aimed to gain new insights into the still poorly understood role of CSA outside TC-NER. To this purpose, in previous work, our laboratory has generated a cell line expressing at physiological levels the wild-type CSA protein (wtCSA) fused *in frame* at its C-terminus with the Flag and HA epitope tags (CSA^Flag-HA^) (Fig. S1). By the tandem affinity purification (TAP) followed by mass spectrometry, the identity of the CSA-interacting proteins was determined, 47 CSA-interacting proteins, 11 of which are involved in TC-NER while the remaining 36 appeared as novel interactors (Lanzafame et al., *in preparation*). Three subunits (namely, CCT3, CCT8, and TCP1) of the TRiC/CCT, which is a chaperonin complex involved in the folding of newly synthesized or unfolded proteins, are novel CSA-interactors. The interaction between CSA and the CCT3, CCT8, and TCP1 subunits has been extensively investigated in the laboratory (Uggè et al., *in preparation*). TRiC/CCT is a complex structure formed by eight CCT subunits (CCT1-CCT8). The CCT1 subunit is more generally known as TCP1 (T-complex protein 1). TRiC/CCT is involved in the folding of proteins characterized by complex topologies and regions of β-strand, among which are WD repeat proteins [17–19]. Just a few proteins have been known as TRiC/CCT substrates, including some cell cycle-related proteins [20], the cytoskeletal proteins, and the telomerase cofactor TCAB1 [21]. To investigate whether specific CSA mutations may affect the CSA/TRiC interaction, our laboratory has generated, using the recombinase-mediated cassette exchange (RMCE) technique, a panel of isogenic cell lines expressing mutated forms of the CSA^Flag-HA^ protein. The selected mutations hit different WD domains: in particular, the E52V amino acidic change (E52V-CSA^Flag-HA^) resides in the first WD repeat (WD1), the Q106P substitution (Q106P-CSA^Flag-HA^) modifies WD2, whereas the K174A change (K174A-CSA^Flag-HA^) maps at the end of WD3 (Fig. S1). The isogenic cell lines were obtained from the CS-A defective CS3BE cells (CS3BE-CSA^Flag-HA^) (Fig. 1). The present work is directed to investigate by immunofluorescence analysis the impact of the selected CSA mutations on the CSA subcellular localization. In addition, we investigate whether the CSA mutations may influence the subcellular localization of TRiC subunits or the CSA/TRiC interaction.

## Methods

### Human cells

The study was performed on SV40-transformed human fibroblasts (CS3BE) isolated from a CSA-defective (CS-A) patient. The CS3BE-derivative cell lines (isogenic cell lines) were previously obtained in the laboratory (Table S1) and contain a single copy of either *cassette1* (pLNeoTkL2) or *cassette2*. *Cassette1* includes the selectable markers neomycin resistance and *HSV-TK* (herpes simplex virus 1 thymidine kinase), whereas *cassette2* includes the selectable marker puromycin resistance and the cDNA encoding the wild type or one of the mutated forms of CSA (E52V, Q106P, or K174A) tagged with Flag-HA (Table S1).

### Culture conditions

Cells were routinely cultured at 37 °C in a humidified atmosphere conditioned with 5% CO_2_. SV40-transformed cell lines were grown in Dulbecco’s Modified Eagle Medium (DMEM, EuroClone) supplemented with 10% fetal bovine serum (FBS, Gibco by Life Technologies), 2 mM l-glutamine (EuroClone), 0.1 mg/ml streptomycin (EuroClone), and 100 U/ml penicillin (EuroClone). In addition, the medium used for the CS3BE-cassette1 cell line was supplemented with 250 μg/ml geneticin (G418, Gibco), whereas that for the CS3BE-cassette2 cell lines was supplemented with 0.15 μg/ml puromycin (Life Technologies). Cells were routinely tested for a mycoplasma-free environment. Sub-culturing was performed by trypsinization and dilution. Briefly, cells are washed with sterile PBS (phosphate buffer saline) and subsequently incubated for 2-3 min with trypsin containing solution (0.05%). Detached cells are resuspended and diluted 1:10 in complete fresh media, and plated in new Petri dishes. For cell preservation and storage, aliquots were trypsinized, centrifuged at 1800×*g* for 8 min, resuspended in 1 ml culture medium containing 10% dimethylsulfoxide (DMSO, Sigma), maintained for 24 h at −80 °C in a suitable box containing isopropanol (Cryostep, Nalgene) and then stored in liquid nitrogen at −196 °C. To establish a new cell culture, a stored aliquot of the cell line of interest was thawed. To avoid the cytotoxic effect of DMSO, cells were rapidly diluted in 7 ml of complete culture medium and seeded in a new culture dish. All the solutions, cell media and the material used for cell cultures were sterile.

### Immunofluorescence

For immunofluorescence analysis, CS3BE-cassette1, CS3BE-wtCSA^Flag-HA^, CS3BE-E52V-CSA^Flag-HA^, CS3BE-Q106P-CSA^Flag-HA^, and CS3BE-K174A-CSA^Flag-HA^ cells were seeded in multi-well tissue culture plates (24 well), each well containing 12 mm sterilized coverslips. After growing in standard conditions for 2 days, yielding about 80% of confluence, cells were processed. The multi-well containing the coverslips was placed on ice, the culture medium was removed and the cells were washed with PBS. Next, cells were fixed for 12 min by incubation with a solution of 3.7% paraformaldehyde (PFA) in PBS. After fixation, cells were washed with PBS for 2 min and then permeabilized in PBS containing 0.1% Triton X-100 for 2 min. The permeabilization was followed by two washes in PBS for 5 min and one extra wash in PBS containing 0.05% Tween (PBS-T) for another 5 min.

To avoid unspecific binding of the primary antibody, cells were first incubated for 30 min with 20 μl of blocking solution (PBS-T containing 5% BSA) in a wet chamber. The wet chamber was settled by placing paper embedded with PBS at the bottom of a closed plastic box to maintain humidity during the entire period of hybridization. After the blocking step, cells were incubated for 1 h with 20 μl of the blocking solution containing suitable dilution of the primary antibodies (see Table S2). For the subcellular localization analysis of CCT3, CCT8, and TCP1 subunits, we used primary antibodies that were raised in rabbit, for the recombinant CSA^Flag-HA^ proteins, we used anti-HA antibodies that were raised in rat (Table S2). For co-localization analysis, cells were incubated with a solution containing two primary antibodies, the anti-HA and the anti-TCP1 or anti-CCT8. Following incubation with the primary antibody, the coverslips were transferred back to the multi-well tissue culture plates and washed three times in PBS-T for 5 min. The incubation with the proper secondary antibody and the washing steps were carried out as above. For the subcellular localization analysis, we used anti-rabbit or anti-rat antibodies conjugated with the Alexafluor 488 or the Alexafluor 555 fluorescent dyes, respectively. For the co-localization analyses, both secondary antibodies were utilized in the same solution. Nuclei were counterstained by incubating the cells for 10 min in PBS containing 0.2 μg/ml of DAPI (4′,6-diamidino-2-phenylindole). Cells were washed three times in PBS and the coverslips were then mounted on slides with Mowiol 4-88 (Calbiochem, 2.4 g Mowiol, 6 g glycerol, 12 ml 0.2 M Tris pH 8.5, 6 ml water). Mowiol was let to dry at room temperature overnight and afterwards, the slides were stored at −20 °C. Immunofluorescence preparations were analyzed on an Olympus IX71 inverted microscope equipped with a digital CCD camera (Robert Scientific Photometrics) and Bandpass filters Omega Optical according to the excitation and emission wavelength of the fluorescent days (Table S3). Images were acquired using the MetaMorph software.

## Results

### Subcellular localization of wild type and mutated forms of the recombinant CSA^Flag-HA^ protein

The subcellular localization of the recombinant CSA^Flag-HA^ protein, either in its wild type (wtCSA^Flag-HA^) or mutated forms (E52V-, Q106P-, or K174A-CSA^Flag-HA^), was investigated by immunofluorescence with antibodies raised against the HA epitope tag (anti-HA). The analysis was performed on the isogenic cell lines previously generated: the CS3BE-wtCSA^Flag-HA^, CS3BE-E52V-CSA^Flag-HA^, CS3BE-Q106P-CSA^Flag-HA^, CS3BE-K174A-CSA^Flag-HA^, and CS3BE-cassette1 cells, the last ones serving as negative control (Fig. 1). We found that the wtCSA^Flag-HA^ protein is mainly localized in the nucleus with a less intense, but not omitted, staining inside the nucleolus, in agreement with previous observations [22, 23]. Differently, CSA^Flag-HA^ proteins with the single amino acid change E52V or Q106P localized mainly in the cytoplasmic and perinuclear region, while the CSA^Flag-HA^ protein carrying the K174A substitution equally distributed in the cytoplasmic and nuclear compartments with a clear accumulation in nucleoli. Overall, these results revealed that single amino acid substitutions affect the subcellular localization of the wtCSA^Flag-HA^ and increase its accumulation in the nucleolus.

### Subcellular localization of the TRiC complex in the isogenic cell lines expressing the wild type or mutated forms of CSA^Flag-HA^

Previous studies in the laboratory have shown that CSA interacts with the TRiC chaperonin complex. In particular, it was shown a direct interaction of CSA with the TCP1, CCT3, and CCT8 subunits of the TRiC complex (Uggè et al., *in preparation*). Therefore, we investigated whether amino acids changes in CSA, which affect the cellular localization of the protein itself, may also impact the cellular distribution of the TRiC complex. To address this issue, we looked at the subcellular localization of the complex by immunofluorescence analysis using antibodies raised against the TCP1, CCT3, and CCT8 subunits in the parental cell line CS3BE-cassette1 as well as in all the isogenic cell lines expressing either the wild type or mutated forms of CSA^Flag-HA^. Since all the primary antibodies (anti-TCP1, anti-CCT3, and anti-CCT8) were originated in rabbit, we first performed a control experiment using only the secondary anti-rabbit antibody. As shown in Fig. S2, no fluorescence background signal was observed in CS3BE-wtCSA^Flag-HA^ cells.

Next, we used anti-CCT3 antibody to investigate the cellular localization of this subunit of the TRiC complex. In all the isogenic cell lines, we found that the protein is localized mainly in the cytoplasm and perinuclear region, where protein synthesis and folding occurs. Also, we observed a very faint nuclear staining with occasional fluorescent dots of accumulations. Relevant of note, by comparing the immunofluorescence pattern distribution of CCT3 in the different cell lines, we did not observe any significant alteration. The protein is similarly distributed in CS3BE-cassette1, which lacks a functional CSA protein, and in all the isogenic cell lines expressing either the wild type or the mutated forms of CSA^Flag-HA^ (Fig. S3).

**Fig. 2:**
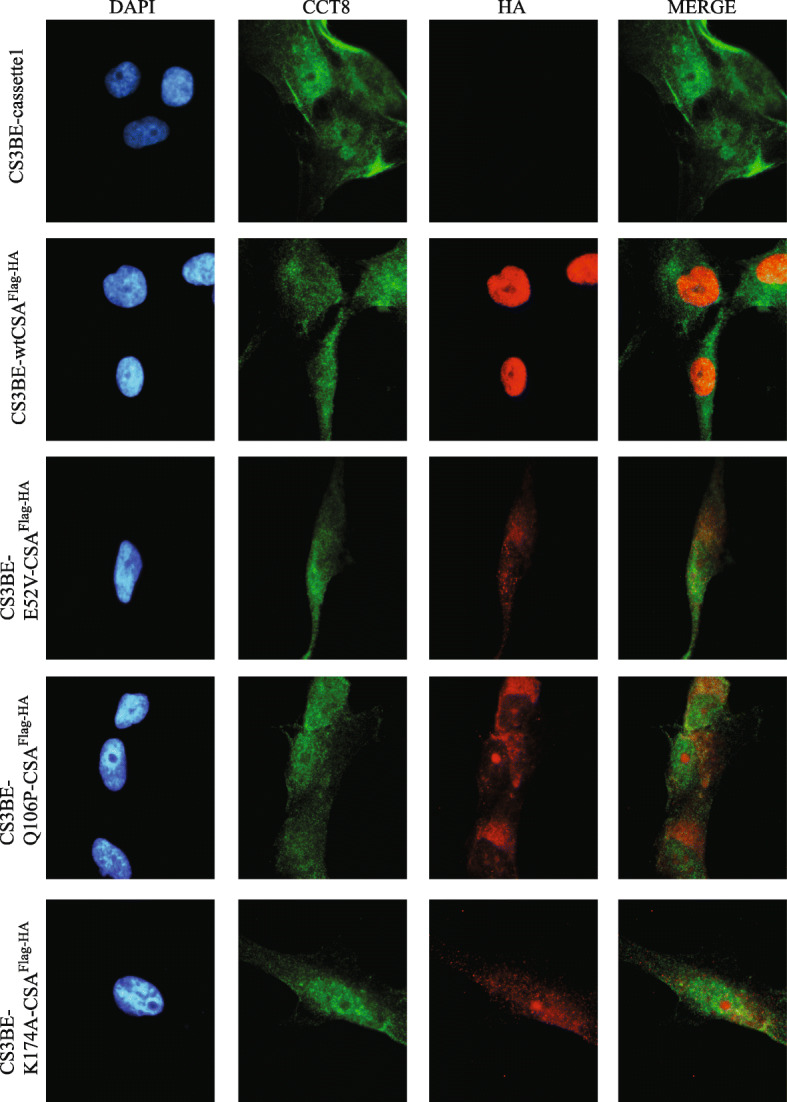
Subcellular co-localization of the recombinant CSA^Flag-HA^ proteins with CCT8 subunit. Double immunofluorescence staining with anti-HA antibodies (*red*) and anti-CCT8 (*green*) in CS3BE-cassette1, CS3BE-wtCSA^Flag-HA^, CS3BE-E52V-CSA^Flag-HA^, CS3BE-Q106P-CSA^Flag-HA^, and CS3BE-K174A-CSA^Flag-HA^. Nuclei were counterstaining with DAPI (*blue*)

Antibodies raised against the CCT8 subunit of the TRiC complex revealed that CCT8 shows a similar cellular distribution as CCT3. Also, this subunit of the TRiC complex localizes mainly in the cytosol and perinuclear region, with no evident exclusion from the nuclear compartment (Fig. 2; S4). Moreover, similarly to CCT3, no alterations were observed by comparing the immunofluorescence pattern distribution of CCT8 in the different cell lines. Overall, these data allowed us to conclude that the functionality of CSA protein does not impact the subcellular localization of the CCT3 or CCT8 proteins.

**Fig. 3:**
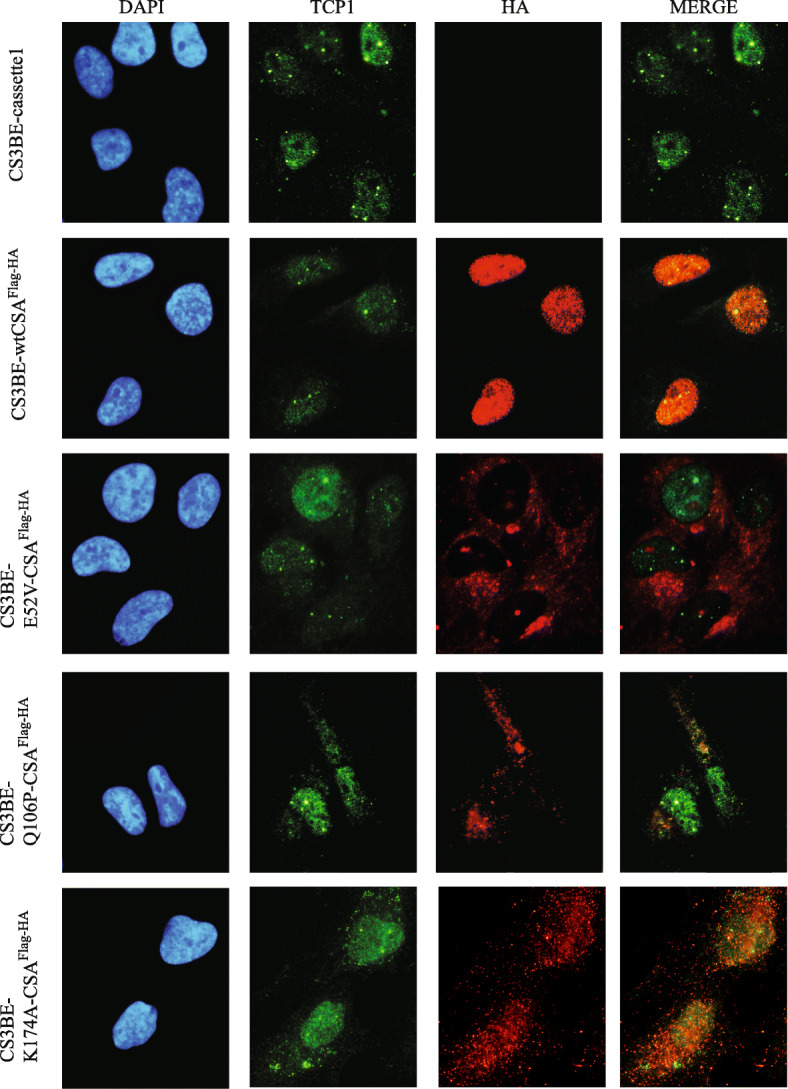
Subcellular co-localization of the recombinant CSA^Flag-HA^ proteins with TCP1 subunit. Double immunofluorescence staining with anti-HA antibodies (*red*) and anti-TCP1 (*green*) in CS3BE-cassette1, CS3BE-wtCSA^Flag-HA^, CS3BE-E52V-CSA^Flag-HA^, CS3BE-Q106P-CSA^Flag-HA^, and CS3BE-K174A-CSA^Flag-HA^. Nuclei were counterstaining with DAPI (*blue*)

Then, we investigated the cellular localization of the TCP1 subunit of the TRiC complex by using anti-TCP1 antibody. We found that this subunit reveals a different cellular distribution compared to CCT3 and CCT8. Indeed, TCP1 is more abundantly present in the nuclei where it tends to accumulate in specific nuclear structures that we will refer to as nuclear bodies (NBs). It is also possible that these structures may represent Cajal bodies (CBs), according to literature data showing that TRiC subunits are required for the proper assembly and trafficking of telomerase to CBs [21]. Once more, by comparing the immunofluorescence pattern distribution of TCP1 in the different cell lines, we did not find major differences either in CS3BE-cassette1 or in the other isogenic cell lines expressing the wild type or mutated forms of CSA^Flag-HA^ (Fig. 3; S5).

### Cellular co-localization of the recombinant wtCSA^Flag-HA^ or the mutated forms of CSA^Flag-HA^ protein with the TRiC complex

As a result of the similarity between CCT3 and CCT8, the cellular co-localization of wtCSA^Flag-HA^ or the mutated forms of CSA^Flag-HA^ with the TRiC complex was further investigated by double immunofluorescence staining with anti-HA and anti-CCT8 or anti-TCP1 antibodies in CS3BE-cassette1 and all the isogenic cell lines. We found that the nuclear wtCSA^Flag-HA^ recombinant protein showed some restricted co-localization signal with the faint nuclear fluorescence staining of CCT8 (Fig. 2), which per se is more abundantly found in the cytoplasm (Fig. 2). Notably, all the mutated forms of wtCSA^Flag-HA^ that tend to accumulate in the cytoplasm (Fig. 2), present a stronger co-localization with the cytoplasmic fluorescence of CCT8 subunit. A different pattern is observed for the TCP1 subunit that it is more abundantly found in the nucleus. The nuclear wtCSA^Flag-HA^ co-localizes with nuclear TCP1 and the TCP1-positive nuclear bodies (NBs) whereas the mutated CSA^Flag-HA^ proteins showed very mild co-localization signals (Fig. 3).

## Discussion

The present work is the follow-up of a previous study directed to identify the functional roles of CSA outside TC-NER and whose deregulation may explain some of CS clinical features. By immunofluorescence analysis, we observed that the recombinant wtCSA^Flag-HA^ localized mainly in the nucleus with weaker but still detectable staining in the nucleolus (Fig. 1), in agreement with previous observations on the endogenous CSA protein [22, 23]. In contrast, the E52V, Q106P, and K174A mutated forms of CSA that our laboratory generated showed a partially or totally altered cellular localization. The selected modifications were engineered with the idea to interfere with the beta-propeller structure of CSA since they map in the WD repeats of the protein. In particular, the E52V amino acid change involves a non-conserved residue on the surface of WD1. The Q106P missense mutation was found in one CS-A patient whose severity was not reported [24]. Since the change involves a conserved amino acid of one of the four beta-sheets in WD2 domain and since the proline residue is known to interfere with the beta-sheets structures of globular proteins [25], it is likely that Q106P impairs the 3D structure of CSA. Finally, the K174A change maps at the end of WD3. The substitution of this amino acid may be at a position less detrimental than Q106P or E52V, thus explaining the fact that the recombinant CSA carrying the K174A change reaches the nuclear compartment more easily than the other two mutants. Overall, we conclude that the occurrence of single amino acid substitutions can alter the subcellular localization of the CSA^Flag-HA^ protein, possibly affecting its nuclear translocation. In a previous work performed in our laboratory, the presence of the Flag and HA tags allowed the purification of the wtCSA^Flag-HA^ interacting proteins and their identification through mass spectrometry (Lanzafame et al., *in preparation*). Thanks to this approach, our laboratory has also found that CSA interacts with the TRiC/CCT complex. Since our laboratory has demonstrated that CSA interacts with the TRiC/CCT complex and also that the previously described amino acid changes strengthen the interaction of CSA with the TRiC/CCT subunits (Uggè et al., *in preparation*). Here, by immunofluorescence analysis, we showed that specific CSA mutations, although affecting the cellular localization of CSA itself (Fig. 1), have no impact on the cellular distribution of TRiC/CCT complex subunits (Figs. 2 and 3; S3; S4; S5). In addition, CSA/TRiC co-localization was not affected by CSA mutations (Figs. 2 and 3). Therefore, the subcellular localization and CSA/TRiC interaction do not rely on the functionality of CSA.

## Conclusions

In this work, we further validated the identification of the TRiC/CCT complex as a novel binding partner of CSA. Moreover, our results support the notion that distinct CSA mutations can alter the subcellular localization of the protein, although not affecting the distribution or interaction behavior of the analyzed TRiC/CCT subunits. Even though further experiments are required to dissect the functional meaning of the newly discovered CSA interactions, this article highlights the involvement of CSA protein in molecular pathways other than TC-NER.

## Supplementary Information

**Additional file 1 **Figure S1. Schematic representation of the CSA^Flag-HA^ proteins and the position of the amino acid changes investigated in this study. WD repeat domains are indicated by alternating colors. Red stars indicate the position of the mutations. W, tryptophan; D, aspartic acid. The CSA protein is tagged at its C-terminus with Flag-HA (Uggè et al., *in preparation*). Figure S2. Establishing the experimental conditions for the subcellular localization analysis of TRiC subunits. Immunofluorescence analysis in the absence of the primary antibody to identify possible background signals due to the secondary anti-rabbit antibody in CS3BE-wtCSA^Flag-HA^ cells. Figure S3. Subcellular localization of the CCT3 subunit of the TRiC complex. Immunofluorescence staining with anti-CCT3 antibodies (*green*) in CS3BE-cassette1, CS3BE-wtCSA^Flag-HA^, CS3BE-E52V-CSA^Flag-HA^, CS3BE-Q106P-CSA^Flag-HA^ and CS3BE-K174A-CSA^Flag-HA^ cells. Nuclei were counterstained with DAPI (*blue*). Figure S4. Subcellular localization of the CCT8 subunit of the TRiC complex. Immunofluorescence staining with anti-CCT8 antibodies (*green*) in CS3BE-cassette1, CS3BE-wtCSA^Flag-HA^, CS3BE-E52V-CSA^Flag-HA^, CS3BE-Q106P-CSA^Flag-HA^ and CS3BE-K174A-CSA^Flag-HA^ cells. Nuclei were counterstained with DAPI (*blue*). Figure S5. Subcellular localization of the TCP1 subunit of the TRiC complex. Immunofluorescence staining with anti-TCP1 antibodies (*green*) in CS3BE-cassette1, CS3BE-wtCSA^Flag-HA^, CS3BE-E52V-CSA^Flag-HA^, CS3BE-Q106P-CSA^Flag-HA^ and CS3BE-K174A-CSA^Flag-HA^ cells. Nuclei were counterstained with DAPI (*blue*). White arrows indicate the accumulation of TCP1 in specific nuclear structures, likely nuclear bodies (NBs). Table S1. CS3BE-isogenic cell lines. Table S2. Antibodies used in this study. Table S3. Fluorochrome excitation and emission wavelength.

## Data Availability

All data generated or analyzed during this study are included in this manuscript and supplementary materials.

## Abbreviations

Ab: Antibody
APS: Ammonium persulfate
ATP: Adenosine triphosphate
BER: Base excision repair
BSA: Bovine serum albumin
CB: Cajal bodies
cDNA: Complementary DNA
CCT: Chaperonin containing TCP-1
CS: Cockayne syndrome
CSA: Cockayne syndrome protein A
CSB: Cockayne syndrome protein B
DAPI: 4′,6-diamidino-2-phenylindole
DMEM: Dulbecco’s Modified Eagle Medium
DMSO: Dimethylsulfoxide
FBS: Fetal bovine serum
GGR: Global genome repair
IF: Immunofluorescence
NBs: Nuclear bodies
NER: Nucleotide excision repair
RMCE: Recombinase-mediated cassette exchange
TAP: Tandem affinity purification
TC-NER: Transcription-coupled nucleotide excision repair
TCP1: T-complex 1
UV: Ultraviolet
WD: Tryptophan-aspartic acid
