# Supplementary material for: Immunofluorescence studies to dissect the impact of Cockayne syndrome A alterations on the protein interaction and cellular localization
Source: J Genet Eng Biotechnol. 2021 Jun 16;19:88. doi: 10.1186/s43141-021-00190-7 (PMC8208330; doi:10.1186/s43141-021-00190-7)
Supplement: Supplementary file 1 — Additional file 1 Figure S1. Schematic representation of the CSAFlag-HA proteins and the position of the amino acid changes investigated in this study. WD repeat domains are indicated by alternating colors. Red stars indicate the position of the mutations. W, tryptophan; D, aspartic acid. The CSA protein is tagged at its C-terminus with Flag-HA (Uggè et al., in preparation). Figure S2. Establishing the experimental conditions for the subcellular localization analysis of TRiC subunits. Immunofluorescence analysis in the absence of the primary antibody to identify possible background signals due to the secondary anti-rabbit antibody in CS3BE-wtCSAFlag-HA cells. Figure S3. Subcellular localization of the CCT3 subunit of the TRiC complex. Immunofluorescence staining with anti-CCT3 antibodies (green) in CS3BE-cassette1, CS3BE-wtCSAFlag-HA, CS3BE-E52V-CSAFlag-HA, CS3BE-Q106P-CSAFlag-HA and CS3BE-K174A-CSAFlag-HA cells. Nuclei were counterstained with DAPI (blue). Figure S4. Subcellular localization of the CCT8 subunit of the TRiC complex. Immunofluorescence staining with anti-CCT8 antibodies (green) in CS3BE-cassette1, CS3BE-wtCSAFlag-HA, CS3BE-E52V-CSAFlag-HA, CS3BE-Q106P-CSAFlag-HA and CS3BE-K174A-CSAFlag-HA cells. Nuclei were counterstained with DAPI (blue). Figure S5. Subcellular localization of the TCP1 subunit of the TRiC complex. Immunofluorescence staining with anti-TCP1 antibodies (green) in CS3BE-cassette1, CS3BE-wtCSAFlag-HA, CS3BE-E52V-CSAFlag-HA, CS3BE-Q106P-CSAFlag-HA and CS3BE-K174A-CSAFlag-HA cells. Nuclei were counterstained with DAPI (blue). White arrows indicate the accumulation of TCP1 in specific nuclear structures, likely nuclear bodies (NBs). Table S1. CS3BE-isogenic cell lines. Table S2. Antibodies used in this study. Table S3. Fluorochrome excitation and emission wavelength. [file 43141_2021_190_MOESM1_ESM.docx]

**Additional File 1**

**Figures**

| 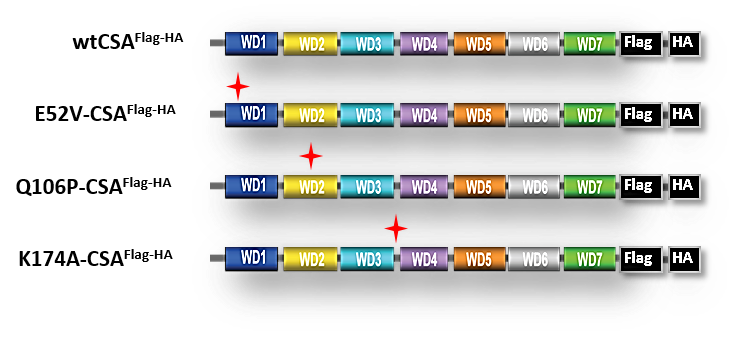 |
| --- |
| **Figure S1. Schematic representation of the CSA^Flag-HA^ proteins and the position of the amino acid changes investigated in this study.** WD repeat domains are indicated by alternating colors. Red stars indicate the position of the mutations. W, tryptophan; D, aspartic acid. The CSA protein is tagged at its C-terminus with Flag-HA (Uggè et al., *in preparation*). |

| 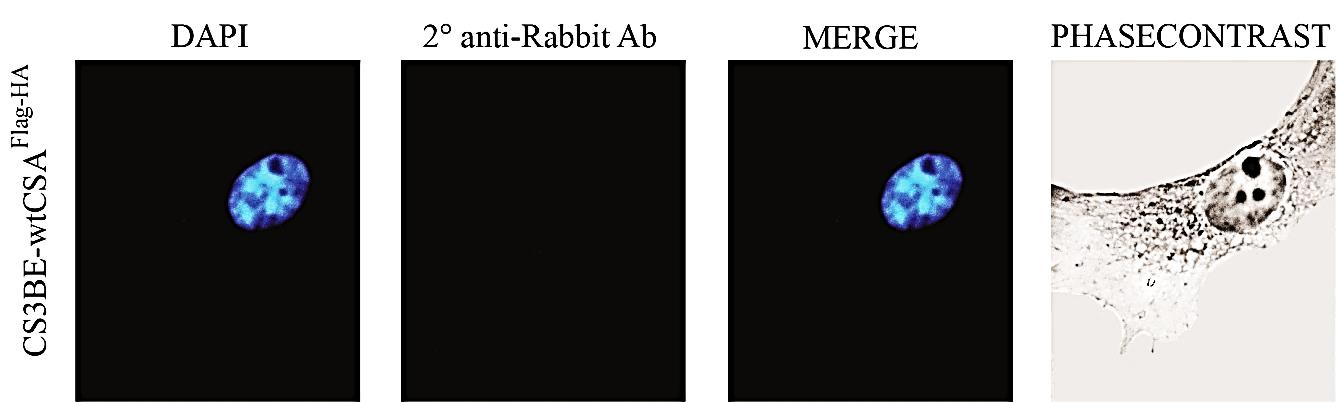 |
| --- |
| **Figure S2. Establishing the experimental conditions for the subcellular localization analysis of TRiC subunits.** Immunofluorescence analysis in the absence of the primary antibody to identify possible background signals due to the secondary anti-rabbit antibody in CS3BE-wtCSA^Flag-HA^ cells. |

| 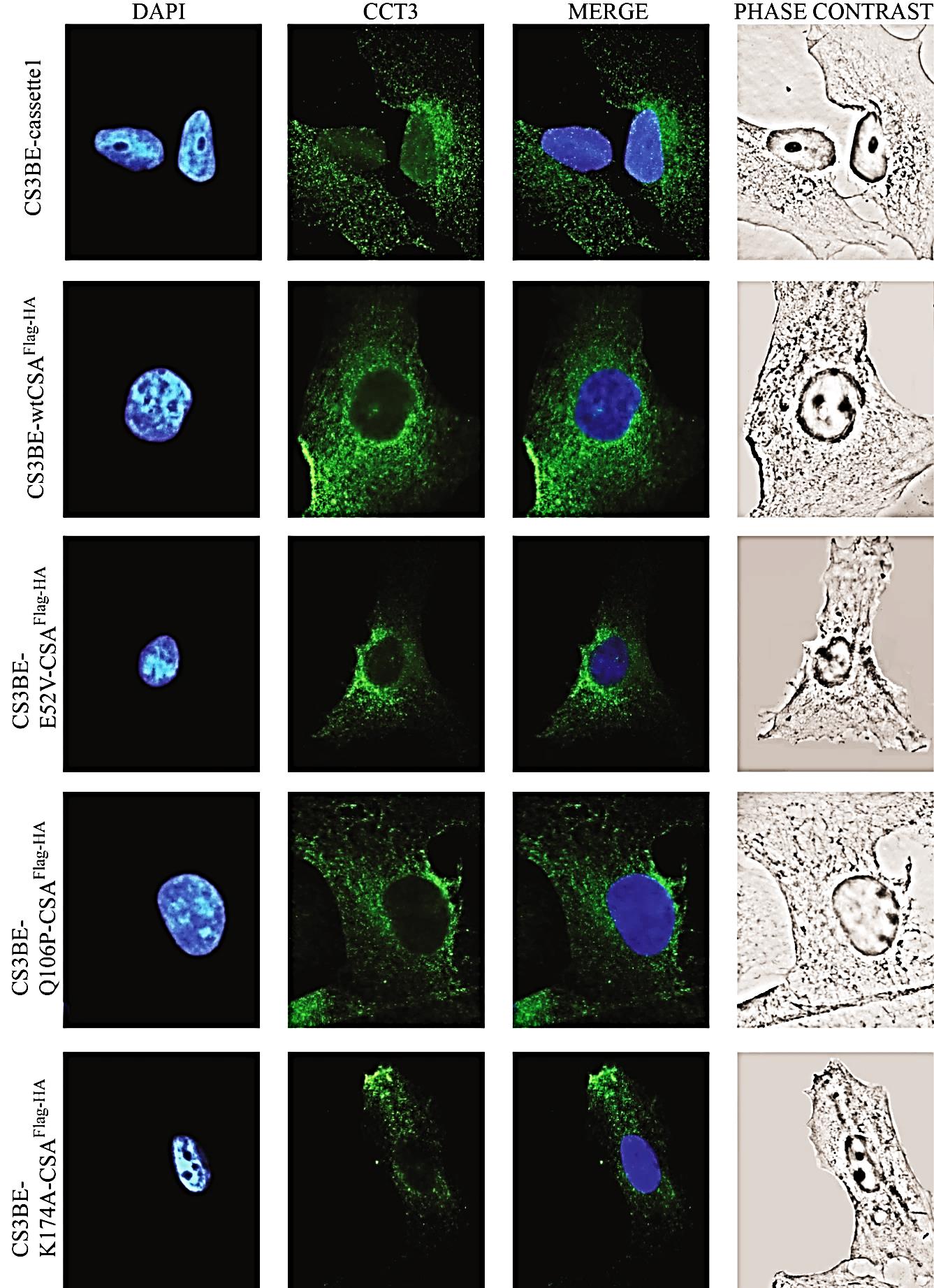 |
| --- |
| **Figure S3. Subcellular localization of the CCT3 subunit of the TRiC complex.** Immunofluorescence staining with anti-CCT3 antibodies (*green*) in CS3BE-cassette1, CS3BE-wtCSA^Flag-HA^, CS3BE-E52V-CSA^Flag-HA^, CS3BE-Q106P-CSA^Flag-HA^ and CS3BE-K174A-CSA^Flag-HA^ cells. Nuclei were counterstained with DAPI (*blue*). |

| 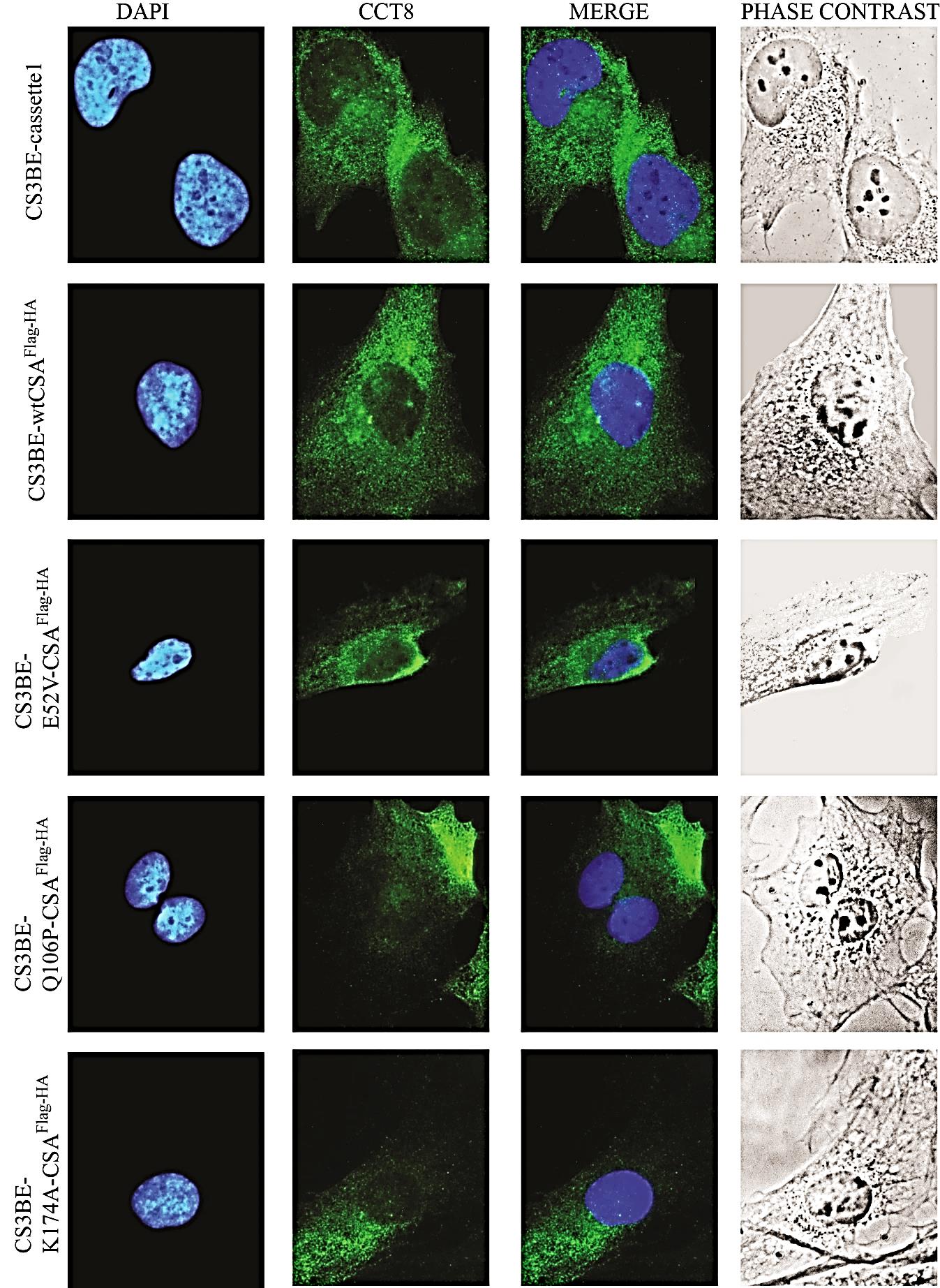 |
| --- |
| **Figure S4. Subcellular localization of the CCT8 subunit of the TRiC complex.** Immunofluorescence staining with anti-CCT8 antibodies (*green*) in CS3BE-cassette1, CS3BE-wtCSA^Flag-HA^, CS3BE-E52V-CSA^Flag-HA^, CS3BE-Q106P-CSA^Flag-HA^ and CS3BE-K174A-CSA^Flag-HA^ cells. Nuclei were counterstained with DAPI (*blue*). |

| ^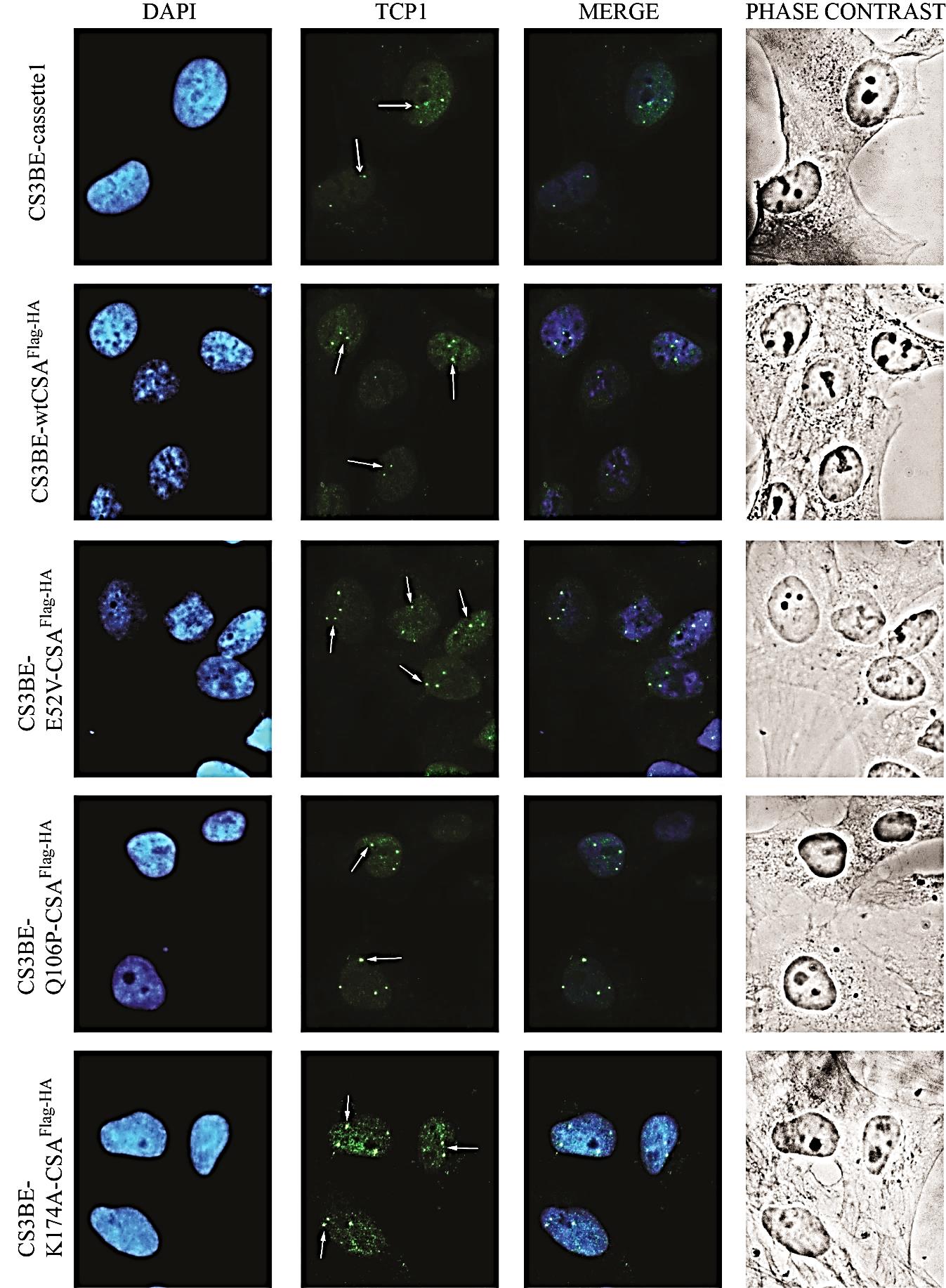^ |
| --- |
| **Figure S5. Subcellular localization of the TCP1 subunit of the TRiC complex.** Immunofluorescence staining with anti-TCP1 antibodies (*green*) in CS3BE-cassette1, CS3BE-wtCSA^Flag-HA^, CS3BE-E52V-CSA^Flag-HA^, CS3BE-Q106P-CSA^Flag-HA^ and CS3BE-K174A-CSA^Flag-HA^ cells. Nuclei were counterstained with DAPI (*blue*). White arrows indicate the accumulation of TCP1 in specific nuclear structures, likely nuclear bodies (NBs). |

**Tables**

| **Table S1 CS3BE-isogenic cell lines.** | |
| --- | --- |
| **Isogenic cell lines** | **Exogenous allele** |
| CS3BE-cassette1 | Containing *cassette1* (pLNeoTK1) |
| CS3BE-wtCSA^Flag-HA^ | Expressing wtCSA^Flag-HA^ |
| CS3BE-E52V-CSA^Flag-HA^ | Expressing E52V-CSA^Flag-HA^ |
| CS3BE-Q106P-CSA^Flag-HA^ | Expressing Q106P-CSA^Flag-HA^ |
| CS3BE-K174A-CSA^Flag-HA^ | Expressing K174A-CSA^Flag-HA^ |

| **Table S2. Antibodies used in this study** | | | |
| --- | --- | --- | --- |
| **Antibody** | **Dilution for IF** | **Origin** | **Reference** |
| Anti-TCP1 | 1:300 | Rabbit | Bethyl, A303-444A |
| Anti-CCT3 | 1:200 | Rabbit | Bethyl, A303-458A |
| Anti-CCT8 | 1:200 | Rabbit | Bethyl, A303-446A |
| Anti-HA | 1:300 | Rat | Roche, 11 867 423 001 |
| AlexFluor 488-conjugated  anti-rabbit IgG | 1:400 | Goat | Jackson Immunoresearch, 111-545-045 |
| AlexFluor 555-conjugated  anti-rat IgG | 1:500 | Goat | Life Technologies A-21434 |

| **Table S3. Fluorochrome excitation and emission wavelength** | | |
| --- | --- | --- |
| **Fluorochrome** | **Excitation Wavelength** | **Emission Wavelength** |
| Alexafluor 488 (green) | 495 nm | 519 nm |
| Alexafluor 555 (red) | 556 nm | 573 nm |
| 4,6-Diamidino-2 Phenylindole (DAPI) | 358nm | 461 nm |
